# Understanding the formation of multiply twinned structure in decahedral intermetallic nanoparticles

**DOI:** 10.1107/S2052252519002562

**Published:** 2019-04-12

**Authors:** Chao Liang, Yi Yu

**Affiliations:** a School of Physical Science and Technology, Shanghaitech University, Shanghai 201210, People’s Republic of China

**Keywords:** intermetallic nanoparticles, multiple twinning, disclinations, aberration-corrected scanning transmission electron microscopy

## Abstract

The structure of monometallic decahedral multiply twinned nanoparticles (MTPs) has been extensively studied, whereas less is known about intermetallic MTPs, especially the mechanism of formation of multiply twinned structures, which remains to be understood. Here, by using aberration-corrected scanning transmission electron microscopy, a detailed structural study of AuCu decahedral intermetallic MTPs is presented. Surface segregation has been revealed on the atomic level and the multiply twinned structure was studied systematically. Significantly different from Au and Cu, the intermetallic AuCu MTP adopts a solid-angle deficiency of −13.35°, which represents an overlap instead of a gap (+7.35° gap for Au and Cu). By analysing and summarizing the differences and similarities among AuCu and other existing monometallic/intermetallic MTPs, the formation mechanism has been investigated from both energetic and geometric perspectives. Finally, a general framework for decahedral MTPs has been proposed and unknown MTPs could be predicted on this basis.

## Abstract 2

The formation of multiply twinned structures in decahedral intermetallic nanoparticles has been explored and a general framework proposed to understand it.

## 1. Introduction   

Nanoparticles (NPs) have received considerable attention for a variety of applications in the fields of energy (Liu *et al.*, 2015 ▸), catalysis (Luo & Guo, 2017 ▸), environment (Nel *et al.*, 2009 ▸) and biology (Gao *et al.*, 2004 ▸). Previous works on NPs including their synthesis (Murphy *et al.*, 2005 ▸; Wiley *et al.*, 2005 ▸), characterization (Sumio, 1987 ▸; Polte *et al.*, 2010 ▸), properties (Katz & Willner, 2004 ▸) and practical applications (McFarland & Van Duyne, 2003 ▸) have primarily focused on single-element systems. In recent years, multi-elemental NPs have received great attention because of the wide tunability of their structures and properties leading to applications such as electrocatalysis (Zhang *et al.*, 2014 ▸; Kim *et al.*, 2014 ▸; Feng *et al.*, 2018 ▸; Zhan *et al.*, 2017 ▸; Prabhudev *et al.*, 2013 ▸). Owing to their structural diversity, understanding the microstructure of multi-elemental NPs is crucial for materials design and interpretation of performance.

It has been a great challenge for more than a century to understand the equilibrium shape of NPs since the first analysis by Wulff (1901 ▸). Besides simple cubes and spheres, NPs can exist in many complex morphologies such as octahedra (Niu *et al.*, 2009 ▸), dodecahedra (Liu *et al.*, 2006 ▸), decahedra (Johnson *et al.*, 2008 ▸) and icosahedra (Wang *et al.*, 2009 ▸; Pohl *et al.*, 2014 ▸). From a crystalline point of view, multiply twinned particles (MTPs) are commonly observed together with single-crystal NPs. When it comes to the microstructure of multi-element NPs, its study becomes more difficult owing to their structural and compositional complexity. First of all, precise atomic scale elemental distribution is difficult to characterize (Wang *et al.*, 2009 ▸). Second, there is the possibility of unique NP architectures with the introduction of another element (Wang *et al.*, 2009 ▸; Pohl *et al.*, 2014 ▸).

Single-element based MTPs have been studied extensively (Sumio, 1987 ▸; Bagley, 1965 ▸; Wit, 1972 ▸; Ino, 1966 ▸; Yu *et al.*, 2017 ▸; Marks *et al.*, 1986 ▸, 1994 ▸; Dundurs *et al.*, 1988 ▸). Theoretically, filling the space with multiply twinned sub-crystals will leave a gap of a fixed size (Ino, 1966 ▸), resulting in the formation of an inner-strained field (Johnson *et al.*, 2008 ▸). For the well known face-centered cubic (f.c.c.) metals such as Au and Cu, the gap angle is +7.35°. Bimetallic MTPs that are fully disordered still maintain an f.c.c. structure, whereas for intermetallic MTPs with a face-centered tetragonal (f.c.t.) structure, this structural characteristic is not well known. To date, only a few works have investigated the crystal structure of intermetallic MTPs (Hu *et al.*, 2009 ▸; Li *et al.*, 2014 ▸). Furthermore, theoretical work can be limited when using approximate models without accurate lattice parameters (Peng *et al.*, 2015 ▸). Therefore, to expand our understanding of MTPs to multi-elemental systems, structural characterization of intermetallic MTPs at the atomic level and a comparison with their monometallic counterparts is needed.

In the present work, using aberration-corrected scanning transmission electron microscopy (AC-STEM), we provide a detailed structural analysis of intermetallic AuCu MTPs. A chemically ordered AuCu core encapsulated within a few atoms thick Au shell forms a decahedral structure. Significantly different from pure Au or Cu decahedral MTPs, the intermetallic AuCu decahedral MTP adopts a solid-angle deficiency of −13.35°, which represents an overlap instead of a gap. The formation mechanism has been explained from both energetic and geometric perspectives, and a general framework for both pure metal and intermetallic MTPs is proposed.

## 2. Experimental   

The transmission electron microscopy (TEM) morphology of the NPs was characterized on a JEOL 2100-Plus under 200 kV. Energy-dispersive X-ray spectroscopy (EDS) mapping was obtained on an FEI Titan 60–300. The AC-STEM imaging and STEM-EELS were carried out using an aberration-corrected TEAM0.5 microscope. Powder X-ray diffraction (XRD) patterns were obtained using a Bruker AXS D8 Advance diffractometer with a Co *K*α source (λ_Co *K*α_ = 1.79 Å). Image simulation was performed with the *PRISM* algorithm (Ophus, 2017 ▸).

## 3. Results and discussion   

### 3.1. Characterization of intermetallic AuCu NPs   

**Figure 1:**
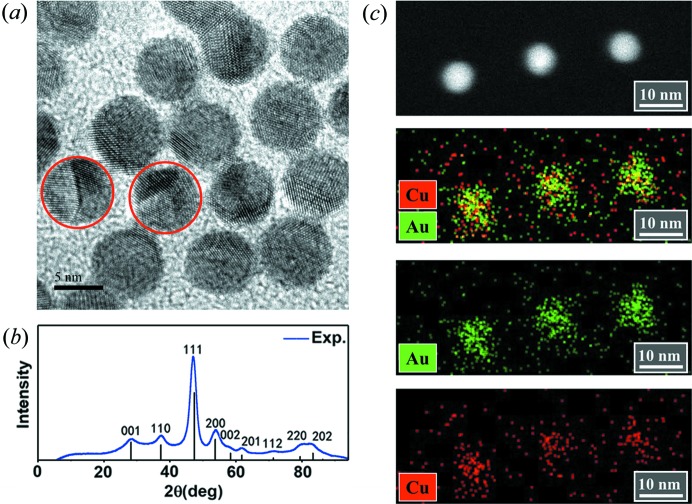
Morphology and elemental distribution of intermetallic AuCu NPs. (*a*) Low-magnification TEM of AuCu intermetallic NPs with a 6 nm diameter. Two MTPs are indicated by red circles. (*b*) XRD and (*c*) STEM-EDS mapping of intermetallic AuCu NPs.

Intermetallic AuCu NPs were successfully synthesized by the seed-mediated technique (Murphy *et al.*, 2005 ▸). Fig. 1 ▸(*a*) shows a TEM image of AuCu NPs with an average diameter of 6 nm. Powder XRD [Fig. 1 ▸(*b*)] shows characteristic diffraction peaks of the intermetallic AuCu (JCPDS 25–1220) phase. Moreover, elemental analysis by EDS [Fig. 1 ▸(*c*)] shows that both Au and Cu are uniformly distributed in a single NP.

**Figure 2:**
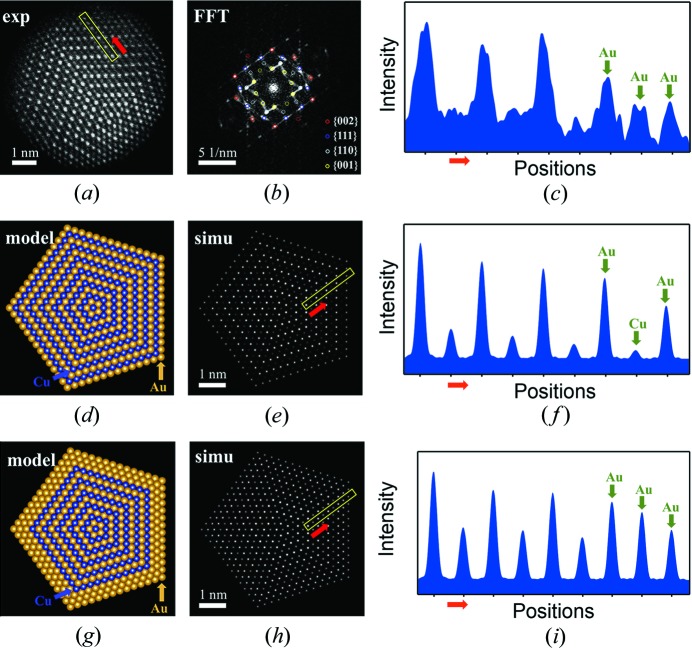
AuCu intermetallic MTPs and surface Au-enriched shell. (*a*) Experimental atomic resolution HAADF image of an AuCu MTP and its FFT in (*b*). Four sets of points are marked with different colors separately and some less obvious points are marked with broken circles. (*c*) Intensity profiles across the particles measured along the direction of the red arrow from the yellow box shown in (*a*). (*d*) Theoretical model of AuCu MTP without a surface Au-enriched shell, its simulated HAADF image in (*e*) and corresponding intensity profile in (*f*). (*g*) Theoretical model of AuCu MTP with a surface Au-enriched shell and its simulated HAADF image in (*h*) and corresponding intensity profile in (*i*).

Apart from single-crystal AuCu NPs, we have also found decahedral AuCu NPs with multiply twinned structures, as indicated by red circles in Fig. 1 ▸(*a*). An atomic resolution image of an intermetallic AuCu MTP is shown in Fig. 2 ▸(*a*). The contrast of the high-angle annular dark-field STEM (HAADF-STEM) image arises from the scattering electrons, which vary with the square of the atomic number *Z*. Therefore, compared with the Cu atom columns (*Z* = 29), the Au atom columns with a larger atomic number (*Z* = 79) show higher image intensity. Furthermore, the clear image contrast shows that the atom columns are perfectly aligned with the incident beam. Oscillatory contrast variations inside the particle illustrate the chemically ordered state of Au and Cu atoms. The fivefold twinned structure can also be easily distinguished, and interestingly, this MTP shows an obvious eccentricity of the fivefold twinned center. Fast Fourier transformation (FFT) was employed to verify its structure. As shown in Fig. 2 ▸(*b*), four groups of points are marked with different colors that indicate different sets of planes. Some spots that are not clearly distinguishable are marked with broken circles and are the result of the small size of the bottom right crystal grain of the AuCu MTP. Again, the FFT patterns further confirm the fivefold twinned structure.

### 3.2. Surface segregation of Au atoms   

The surface atomic structure of NPs is difficult to analyze using conventional TEM owing to the delocalization of the contrast. Taking advantage of AC-STEM, we probed the surface of AuCu MTPs at the atomic scale. As the thickness of the edge of a spherical NP should be smaller, a weaker contrast is normally expected on the edge. However, as shown in the yellow box of Fig. 2 ▸(*a*) and its corresponding intensity profile in Fig. 2 ▸(*c*), atom columns at the outer edge are brighter than the nearest neighbor Cu atom columns. This non-oscillatory contrast at the outer edges has aroused our attention; the presence of the heavy element Au was expected in these atom columns.

To obtain a better understanding, we created different structural models and compared the corresponding simulated images with the experimental one. Figs. 2 ▸(*d*) and 2(*g*) show the two models of AuCu MTPs employed, *i.e.* fivefold twinned AuCu NP without and with a Au-rich surface shell, respectively (see Fig. S1 of the supporting information for details of the models). Figs. 2 ▸(*e*) and 2(*h*) are the simulated images where the Au-rich shell shows a relatively stronger contrast. Figs. 2 ▸(*c*), 2(*f*) and 2(*i*) are the intensity profiles measured along the red arrows as shown in Figs. 2 ▸(*a*), 2(*e*) and 2(*h*), respectively. The major difference arises from the outer three atomic layers and the experimental intensity profile [Fig. 2 ▸(*c*)] agrees well with the model structure that contains the Au-rich shell. This replacement of the original order of Au–Cu–Au by an Au-rich surface of the AuCu NPs could also be observed from the single-crystal AuCu NPs (Fig. S2). Moreover, the existence of the Au-rich shell has also been confirmed by electron energy loss spectroscopy (EELS) (Fig. S3). Therefore, we found that the intermetallic AuCu NPs tend to develop an Au-enriched shell at the surface, which may be critical in their applications such as electrocatalysis (Kim *et al.*, 2017 ▸). This surface segregation could be a general phenomenon as observed in FePt MTPs (Li *et al.*, 2014 ▸; Wang *et al.*, 2008 ▸) and predicted in the Au–Ag system (Peng *et al.*, 2015 ▸).

### 3.3. Solid-angle deficiency in intermetallic decahedral MTPs   

It has been more than fifty years since Ino first proposed the space imperfection of decahedral MTPs (Ino, 1966 ▸). Since then, almost all of the research has focused on MTPs composed of a single element, either metals (Johnson *et al.*, 2008 ▸; Elechiguerra *et al.*, 2006 ▸) or semiconductors (Sumio, 1987 ▸; Yu *et al.*, 2017 ▸). However, the situation of intermetallic MTPs is less well known and the structural difference between pure-metal and intermetallic MTPs remains to be understood.

**Figure 3:**
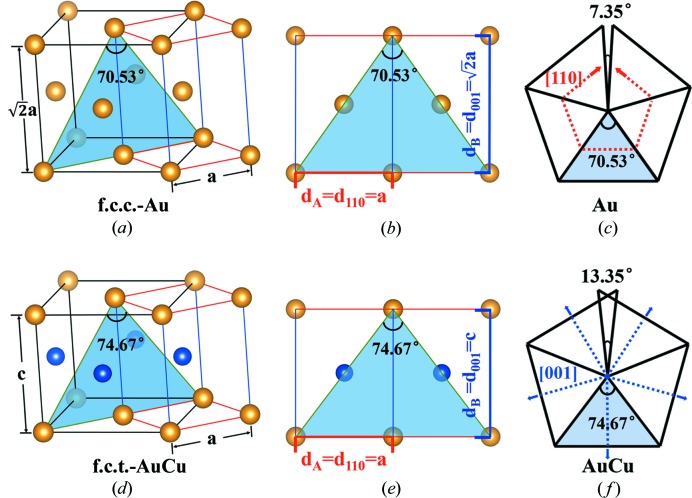
Space imperfection in different decahedral MTPs. (*a*) A unit cell of Au single crystal with f.c.c. structure. A b.c.t. repeating unit can be extracted from the f.c.c. structure. (*b*) Structural projection with a blue triangle which corresponds with a segment of decahedral MTPs. (*c*) Diagram of Au decahedral MTP comprising a gap of 7.35°. The blue triangles with 70.53° angles of the top three images have a one to one correspondence. The main expansion direction [110] has been marked with red broken lines. (*d*) A unit cell of an AuCu single crystal with an f.c.t. structure. A b.c.t. repeating unit can be extracted from the f.c.t. structure. (*e*) Structural projection with a blue triangle that corresponds with a segment of intermetallic decahedral MTPs. (*f*) Diagram of AuCu decahedral MTP comprising an overlap of 13.35°. The blue triangles with 74.67° angles of the bottom three images have a one to one correspondence. The main expansion direction [001] has been marked with blue broken lines.

In order to better illustrate the intrinsic differences between the two, crystal models have been drawn as shown in Fig. 3 ▸. The unit cell of f.c.c. Au is shown in Fig. 3 ▸(*a*), from which one can also extract a repeating unit (outlined with red and blue lines) that can be considered as a special body-centered tetragonal (b.c.t.) cell, with 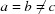, 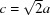. Here, *a*, *b* and *c* are the lattice parameters of the b.c.t. cell. A fivefold twinned decahedral NP can be considered as five tetrahedrons connected together and the direction along the fivefold axis is the 〈110〉 axis of the f.c.c. structure. The projected {110} plane is drawn in Fig. 3 ▸(*b*) and it is also the {100} plane of the b.c.t. cell. Here, we denote 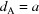 and 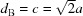 so that the angle highlighted in both Figs. 3 ▸(*a*) and 3(*b*) can be calculated as 2arctan(*d*_A_/*d*_B_) = 70.53°. This is exactly the vertex angle of one of the segments in the fivefold twinned NP. In this way, continuous adhesion of five segments results in a gap of 7.35° as shown in Fig. 3 ▸(*c*). This model is applicable to all f.c.c. mono-elemental decahedral MTPs.

For intermetallic decahedral MTPs, the situation can be different. Taking AuCu as an example, as shown in Fig. 3 ▸(*d*), the crystal has an f.c.t. structure (also known as the L_10_ phase), with Au atom layers and Cu atom layers separated along the *c* axis. The corresponding b.c.t. cell is also illustrated with the red and blue lines, and in the same manner, the projected plane along the fivefold axis of the MTP is shown in Fig. 3 ▸(*e*). In general, *d*_A_ = *a* and *d*_B_ = *c* still applies, however, *d*_A_ and *d*_B_ do not have a specific relationship. In the case of AuCu, the vertex angle is calculated to be 2arctan(*d*_A_/*d*_B_) = 74.67°. Interestingly, this means continuous adhesion of five segments will cause an overlap of 13.35°, as shown in Fig. 3 ▸(*f*).

In this comparison, we can see that the *d*_A_/*d*_B_ value is fixed for the f.c.c. structure (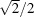), corresponding to a missing gap of 7.35° in the MTPs. In contrast, for the non-f.c.c. intermetallic decahedral MTPs, *d*_A_/*d*_B_ does not have a fixed general value so that the vertex will change depending on the elements combined. Therefore, various gaps and overlaps can occur with the space-filling procedure.

Then we move on to understand how the spaces are actually filled for both cases. For the solid-angle deficiencies in f.c.c. pure metals, the concept of disclination has been introduced and widely adopted (Wit, 1972 ▸). A disclination represents a wedge force moment which removes the deficiency in an MTP. Two edge planes of the gap are brought together to fill the space and a twin boundary is formed. As reported by Wit (1972 ▸), the Au MTP with a gap of 7.35° requires a disclination of +7.35° to fill the gap (Wit, 1972 ▸). This wedge disclination is a partial disclination as its rotation angle of +7.35° and is not a symmetry rotation of the Au lattice. Similarly, the concept of wedge disclination could also be extended to intermetallic MTPs. In the above case, the AuCu MTP requires a disclination of −13.35°. Here, a positive disclination corresponds to closing the gap and a negative disclination corresponds to removing the overlap.

### 3.4. Formation mechanism of intermetallic decahedral MTPs from an energetic perspective   

**Figure 4:**
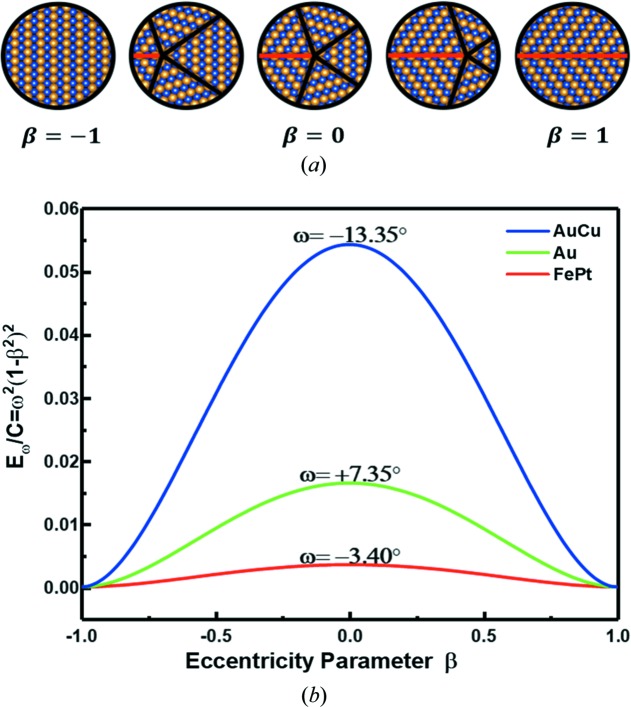
Eccentricity and energy barrier of disclination. (*a*) Diagram of small particles with different eccentricity which can be evaluated by parameter β ranges from −1 to 1. (*b*) Curves of the disclination energy barrier with different eccentricity of different MTPs.

Based on the theoretical framework of elastic energy of disclination (Marks *et al.*, 1986 ▸; Dundurs *et al.*, 1988 ▸), we investigated the formation mechanism of intermetallic decahedral MTPs from an energetic perspective. Their formation is the result of competition between their surface energy and the elastic energy associated with the wedge disclination. The total energy can be expressed as
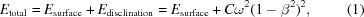
where *C* is a constant for an NP of a particular size and element, ω is the rotation angle of the disclination and β is the degree of eccentricity. As shown in Fig. 4 ▸(*a*), the parameter β, which ranges from −1 to 1, is adopted to evaluate the eccentricity of decahedral MTPs. When there is no eccentricity in the MTPs, β = 0. When the fivefold axis is not located in the center of the MTP, β will have a non-zero value. The larger the eccentricity, the larger the absolute β value. In the extreme case of β = −1, this is a single crystalline NP while in the other extreme case when β = 1 there is one twin boundary across the NP.

To form a specific decahedral MTP, the elastic energy caused by disclination must be overcome. Fig. 4 ▸(*b*) shows plots of the elastic energy of disclination *versus* the eccentricity parameter β. Curves of three known decahedral MTPs (Au, AuCu and FePt) with different eccentricities were drawn for comparison. As can be seen for certain β values, the elastic energy varies for different materials with different rotation angles of the disclinations. The larger the rotation angle, the larger the elastic energy. Therefore, decahedral MTPs may not be favored in materials with large rotation angles as the elastic energy barrier becomes higher. For example, Pd and Zn are able to form intermetallics such as PdZn; however, the calculated rotation angle could be as large as −40°, which may hinder the formation of decahedral MTPs. This may explain why only a small number of intermetallic decahedral MTPs have been reported to date. On the other hand, for a specific material the elastic energy can be lowered by a higher degree of eccentricity. For Au and FePt, the effect is trivial. However, it becomes significant for AuCu and may be the source of the eccentricity as observed in Fig. 2 ▸(*a*).

### 3.5. Formation mechanism of intermetallic decahedral MTPs from a geometric perspective   

**Figure 5:**
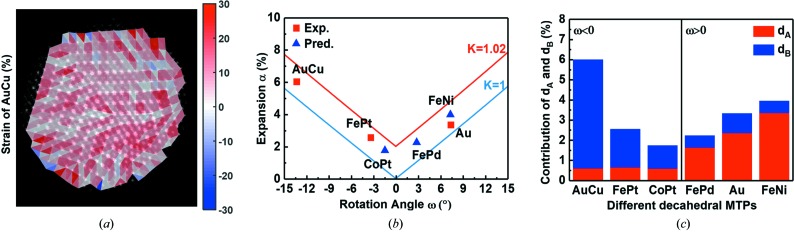
Strain distribution and analysis of lattice distortions. (*a*) Two-dimensional lattice strain map of the AuCu intermetallic decahedral MTP. (*b*) Strain of decahedral MTPs plotted as a function of the disclination rotation angle for both experimental and predicted MTPs. The two lines are the α–ω relationship when *K* = 1.02 (red) and *K* = 1 (blue). (c) Histogram of the *d*_A_ and *d*_B_ contribution to the expansion for both experimental and predicted MTPs.

Geometrically, we have measured the lattice distances and evaluated the strain to further understand the formation mechanism of intermetallic decahedral MTPs. Apart from our AC-STEM imaging of AuCu decahedral MTPs, an aberration-corrected high-resolution TEM (AC-HRTEM) image of Au decahedral MTP (Gautam, 2017 ▸) (see also Fig. S4) was used to serve as a comparison. Figs. S4(*c*) and 5 ▸(*a*) are the strain maps of the Au and AuCu MTPs, respectively. Atomic scale strain mapping was obtained by fitting the individual atom positions (Gan *et al.*, 2012 ▸; Yu *et al.*, 2016 ▸) (see Fig. S5 for details). Lattice expansion and contraction are represented in red and blue, respectively. The strain values are the percentage changes in the lattice relative to the bulk materials. Generally speaking, strain distribution tends to be homogeneous for both cases. In more detail, there is a slight tensile strain concentration at the center of the fivefold axis in an AuCu MTP [Fig. 5 ▸(*a*)]. Interestingly, although there is a clear difference in the solid-angle deficiency of these two materials (gap in Au and overlap in AuCu), both lattices expand on average, indicating both MTPs remove the solid-angle deficiency by lattice expansion.

In order to develop a systematic understanding, experimental data of intermetallic FePt decahedral MTPs (Li *et al.*, 2014 ▸) with the same particle size are also compared in this work. Our experimental data of AuCu, together with the experimental data of Au and FePt from the other two independent reports, were analyzed in the same manner in terms of lattice strain and the results are shown in red cubes in Fig. 5 ▸(*b*), where the percentage expansion is plotted as a function of the rotation angle of disclination. It can be seen that the particles expand regardless of whether the wedge disclination is positive or negative. Meanwhile, the degree of the expansion is positively correlated with the absolute value of the rotation angle of disclination. Here, the AuCu MTPs with the largest absolute value of ω expand the most compared with the other two MTPs.

For further analysis, the lattice expansion can be divided into contributions along the *d*_A_ and *d*_B_ directions (Fig. 3 ▸). The result is shown in Fig. 5 ▸(*c*) and the contributions from the *d*_A_ and *d*_B_ directions are indicated by red and blue columns, respectively. Overall, the unit cell expands along both *d*_A_ and *d*_B_. For Au MTPs with a positive wedge disclination, the cell expands more along the *d*_A_ direction, whereas for AuCu and FePt MTPs with a negative wedge disclination, the cells expand more along the *d*_B_ direction. Such geometric deformation can be understood intuitively through the schematics in Figs. 3 ▸(*c*) and 3(*f*) (the expansions are indicated by arrows). For MTPs with a positive disclination, the particles will fill the gap mainly by expanding along the *d*_A_ direction; for MTPs with a negative disclination, the particles will make room for the redundant wedge by expanding mainly along the *d*_B_ direction.

### 3.6. Prediction of lattice distortions in other potential intermetallic MTPs   

In addition to the above experimental data, other potential intermetallic decahedral MTPs can also be predicted once their formation mechanism has been understood. Analytically, the strain α could be regarded as a function of the rotation angle ω:
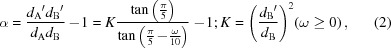


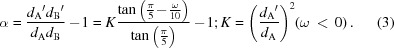
Here, the parameter *K* is related to the lattice expansion along *d*_A_ and *d*_B_, where *d*_A_ and *d*_B_ are the original lattice distances, and *d*_A_′ and *d*_B_′ are the lattice distances after expansion (see the supporting information for a detailed derivation). The above relationship is plotted in Fig. 5 ▸(*b*) for two cases, *i.e.* *K* = 1 and *K* = 1.02 in blue and red, respectively. *K* = 1 corresponds to zero expansion, which is the lower limit (experimentally, the lower limit is supposed to be *K* = 1, though this does not mean that *K* cannot be less than 1). For the upper limit, in principle, there is no limitation from a geometry standpoint. In practice, the lattice should not be able to expand too much, therefore an expansion of several percent could be a good estimation. Here we show the case of *K* = 1.02 with a 1% expansion along the *d*_A_ or *d*_B_ direction.

Within this framework, the formation and lattice strain of FePd, FeNi and CoPt intermetallic decahedral MTPs can be predicted. As discussed, for a specific material, the values of ω, *d*_A_ and *d*_B_ are fixed whereas the values of *d*_A_′ and *d*_B_′ are not unique. In practice, the exact values of *d*_A_′ and *d*_B_′ depend on the situation of strain relaxation in that particular MTP. The experimental (red square) or theoretical (blue triangle) data points in Fig. 5 ▸(*b*) may vary along the vertical axis. As an example, we show the predicted data points with *K* = 1.012 in Figs. 5 ▸(*b*) and 5(*c*) (detailed data of lattice parameters can be found in Table S1). Although specific strain values may vary on a case by case basis, our analytical framework can still provide a guidance as the fluctuation should be restricted within a certain range defined by the lower and upper limits. Furthermore, as we have already seen from the experimental data, the lattice strain should be positively correlated with the absolute value of the rotation angle of disclination. Most importantly, as shown in Fig. 5 ▸(*c*), a universal rule is that the positive and negative rotation angle leads to expansion mainly along *d*_A_ and *d*_B_, respectively. This is applicable for all materials regardless of the specific strain value.

On this basis, the differences and similarities between mono-elemental and intermetallic decahedral MTPs can be fully understood and a general framework could be built. For mono-elemental MTPs with a +7.35° deficiency, the gap is closed by an expansion mainly along the *d*_A_ direction. In this way, the lattice changes from an f.c.c. to an f.c.t. structure from an average perspective. For intermetallic MTPs with an f.c.t. structure, both gaps and overlaps may occur, and the lattice will expand mainly along *d*_A_ or *d*_B_ to make the angle of circumference closer to 360°. In this way, a revised f.c.t. structure could be formed. Additionally, our conclusion about the lattice distortions can also be supported by the experimental measurement in Ag fivefold twinned nanowires (Sun *et al.*, 2012 ▸), further confirming the reliability of our conclusion.

## 4. Conclusions   

We have demonstrated an in-depth atomic scale structural study of AuCu intermetallic decahedral MTPs. The atomically thin Au enriched shells have been verified by using AC-STEM and image simulations. Furthermore, we have explored the differences and similarities between mono-elemental MTPs and intermetallic MTPs. Apart from our experimental AuCu data, we also incorporated the existing data of Au and FePt MTPs into our analysis. From both energetic and geometric perspectives, the formation mechanism of decahedral MTPs was systematically investigated. A general framework for understanding decahedral MTPs has been proposed, and unknown intermetallic MTPs could be predicted on this basis. This could be significant not only in the understanding of existing MTPs but also for designing novel strain-induced intermetallic MTPs for various applications.

## 5. Related literature   

The following references are cited in the supporting information: Chen *et al.* (2010 ▸); Clarke & Scott (1980 ▸); Gan *et al.* (2012 ▸); Gautam (2017 ▸); Johansson & Linde (1936 ▸); Li *et al.* (2014 ▸); Momma & Izumi (2011 ▸); Ophus (2017 ▸); Persson (2016 ▸); Warlimont (1959 ▸); Woolley *et al.* (1964 ▸); Yu *et al.* (2016 ▸); Yuasa *et al.* (1994 ▸).

## Supplementary Material

Supporting information file. DOI: 10.1107/S2052252519002562/zx5018sup1.pdf
