# Supplementary material for: Understanding the formation of multiply twinned structure in decahedral intermetallic nanoparticles
Source: IUCrJ. 2019 Apr 12;6(Pt 3):447–53. doi: 10.1107/S2052252519002562 (PMC6503919; doi:10.1107/S2052252519002562)
Supplement: Supplementary file 1 [file m-06-00447-sup1.pdf]

IUCrJ

Volume 6 (2019)

Supporting information for article:

Understanding the Formation of Multiply-Twinned Structure in Decahedral Intermetallic Nanoparticles

Chao Liang and Yi Yu

Understanding the Formation of Multiply-Twinned Structure in Decahedral Intermetallic Nanoparticles

Chao Liang[a] and Yi Yu[a]*

[a]School of Physical Science and Technology, Shanghaitech University, Shanghai, 201210, People's Republic of China

Correspondence email: yuyi1@shanghaitech.edu.cn

Table of Contents

S1. Material Synthesis
S2. Model
S3. Image Simulation
S4. Electron Energy Loss Spectroscopy (EELS)
S5. Data of Au and FePt
S6. Strain Mapping
S7. Derivation of the Relationship between Strain and Rotation Angle of the Disclination

S1. Material synthesis

Synthesis of Au Seed nanoparticles (NPs). Au seeds were prepared with modified procedures from earlier work (Chen *et al*., 2010). 0.1g of $HAuCl_4 \cdot 3H_2O$ was dissolved in 10 mL of oleylamine and 10 mL of o-xylene at 10 ºC under Ar flow and vigorous magnetic stirring. 0.5 mmol of borane tert-butylamine complex dissolved in 1 mL of oleylamine and 1 mL of o-xylene via sonication was injected into the $HAuCl_4$ solution. After stirring for 1 h at 10 ºC, ethanol was added to precipitate Au NPs via centrifugation. The precipitate was re-dispersed in hexane before addition of ethanol and centrifugation. And the final product was dispersed in hexane again for AuCu intermetallic NP synthesis.

Synthesis of AuCu Chemically Ordered Decahedral NPs. AuCu ordered intermetallic NPs were synthesized with a typical synthesis procedure in a Schlenk line. 0.25 mmol of $Cu(CH_3COO)_2 \cdot H_2O$ was mixed with 0.5 mL of oleic acid and 2.25 mL of tri-n-octylamine in a 25 mL three-neck flask. After heated at 80 ºC for 30 min, the solution became clear and 0.25 mmol of Au NPs dispersed in 20 mL of hexane was added. Once hexane evaporated completely, the mixture was heated at 120 ºC for 20 min under Ar and then quickly heated to 300 ºC for 50 min before cooling down. The product was collected by ethanol addition, centrifugation and re-dispersed in 20 mL of hexane for use.

S2. Model

AuCu intermetallic decahedral multiply-twined particles (MTPs) were constructed with the VESTA software (Momma & Izumi, 2011). As shown in Figure S1, two different models were displayed with both cross-sectional and three-dimensional view. As mentioned in the main text, constructing a AuCu MTP with the original body centered tetragonal (BCT) lattice parameters will cause an overlap. We adjusted the lattice parameters properly based on the rules discussed in the main text to remove the overlap.

S3. Image simulation

STEM image simulations were performed using a plane wave reciprocal space interpolated scattering matrix (PRISM) method (Ophus, 2017). Above mentioned models were taken as input structural models here. The accelerating voltage was 300 kV and interpolation factor was chosen as 8. The probe convergence angle was 17 mrad and collection angle was from 70 to 200 mrad. Spherical aberration C_S was set to 0, considering an aberration-free probe.

For comparison, model constructions and image simulations of a AuCu single-crystal nanoparticle (NP) have also been performed in the same way. The result is shown in Figure S2. The difference in the intensity of outer three layers also demonstrates the existence of gold-enriched shell over the AuCu core as the case of AuCu MTPs.

S4. EELS

STEM-EELS line profiles were performed to demonstrate the existence of the gold enriched shells in AuCu NPs. An example is shown in Figure S3. STEM-EELS line profile was measured along the green arrows' line in Figure S3b and the result was shown in Figure S3a. The EELS counts has been normalized. Au counts were slightly higher than Cu on the edge of the NP. This may support the conclusion drawn from Figure 2 and S2 that gold-enriched shells existed.

S5. Data of Au and FePt

To investigate the effect of different disclination on the atomic structure of MTPs, an aberration-corrected high-resolution TEM (AC-HRTEM) image of a Au MTP (Gautam, 2017) was adopted to serve as a comparison (Figure S4). The image was taken at 600 ºC and its five-fold structure could be observed from both HRTEM image and its FFT. For the FePt, analysis has been performed using the data published before (Li et al., 2014).

S6. Strain mapping

In order to obtain a strain mapping on the atomic-scale, Gaussian fitting of the atom column positions was performed (Yu *et al.*, 2016), and then strain maps were drawn (Gan *et al.*, 2012; Yu *et al.*, 2016). The area of the triangle composed by three Au atom columns was calculated and then converted to strain. As shown in Figure S5a, for the case of Au, each vertex of a triangle corresponds to a gold atom column. While for the case of AuCu in Figure S5b, still the Au-atom-triangle was calculated, and the copper atoms were not counted in owing to the lower contrast which may bring inaccuracy when fitting its position. The area of each triangle (A(x_1,y_1), B(x_2,y_2), C(x_3,y_3)) was calculated with the formula:

$$S = \frac{1}{2}(x_1y_2 + x_2y_3 + x_3y_1 - x_1y_3 - x_2y_1 - x_3y_2)$$

where S is the area. Strain was calculated with the following formula:

$$\text{Percentage Local Strain} = \frac{S - S_0}{S_0} \times 100$$

where the S_0 represents the standard area in single-crystal NPs. The value of the overall strain could be obtained by the following formula:

$$\text{Percentage Overall Strain} = (\frac{\overline{d_{\boldsymbol{A}}} \times \overline{d_{\boldsymbol{B}}}}{d_{\boldsymbol{A0}} \times d_{\boldsymbol{B0}}} - 1) \times 100$$

where the $\overline{d_{\boldsymbol{A}}}$ and $\overline{d_{\boldsymbol{B}}}$ corresponds to the arithmetical mean value of d_A' and d_B', respectively. d_A' and d_B' are the expanded lattice parameters measured in each BCT cell.

S7. Derivation of the relationship between strain and rotation angle of the disclination

Table S1 lists the standard lattice parameters and the rotation angles of the disclination of several materials (Johansson & Linde, 1936; Yuasa *et al.*, 1994; Warlimont, 1959) that were discussed in this

manuscript. The experimental data for Au, FePt, and AuCu MTPs, as well as the predicted CoPt, FeNi, and FePd MTPs are also listed. The chosen of the latter three materials for prediction is because that intermetallic phase of FePd (Persson, 2016), FeNi (Clarke & Scott, 1980), CoPt (Woolley *et al.*, 1964) have been reported before. As can be seen, the angles of circumference would become closer to 360º after lattice expansion. The less expansion in Au MTP could be related to the strain relaxation at high temperature. Derivation is as following:

For the single-crystal NP, we have:

$$tan(\frac{\pi}{5}-\frac{\omega}{10})=\frac{d_{\boldsymbol{A}}}{d_{\boldsymbol{B}}}$$

For the decahedral MTP, we have:

$$tan\frac{\pi}{5}=\frac{d_{\boldsymbol{A}}'}{d_{\boldsymbol{B}}'}$$

By definition, strain can be calculated as:

$$\alpha=\frac{S-S_0}{S_0}=\frac{d_{\boldsymbol{A}}'\times d_{\boldsymbol{B}}'-d_{\boldsymbol{A}}\times d_{\boldsymbol{B}}}{d_{\boldsymbol{A}}\times d_{\boldsymbol{B}}}$$

Here, d_A and d_B are the original lattice distances, and d_A’ and d_B’ are the lattice distances after expansion. Combining the above three formulas, we can derive the relationship in the following. Here, we separate it into two cases, with positive rotation angle and negative rotation angle, respectively.

$$\alpha=\frac{d_{\boldsymbol{A}}'\times d_{\boldsymbol{B}}'}{d_{\boldsymbol{A}}\times d_{\boldsymbol{B}}}-1=\frac{\tan\left(\frac{\pi}{5}\right)}{\tan\left(\frac{\pi}{5}-\frac{\omega}{10}\right)}\times K-1;\;\; K=\left(\frac{d_{\boldsymbol{B}}'}{d_{\boldsymbol{B}}}\right)^2 \;\; (\omega\geq 0)$$

$$\alpha=\frac{d_{\boldsymbol{A}}'\times d_{\boldsymbol{B}}'}{d_{\boldsymbol{A}}\times d_{\boldsymbol{B}}}-1=\frac{\tan\left(\frac{\pi}{5}-\frac{\omega}{10}\right)}{\tan\left(\frac{\pi}{5}\right)}\times K-1;\;\; K=\left(\frac{d_{\boldsymbol{A}}'}{d_{\boldsymbol{A}}}\right)^2 \;\; (\omega< 0)$$

Here, the parameter K is related to the lattice expansion along d_A and d_B directions. For the case of lattice expansion, $K\geq 1$. As discussed in the main text, within certain upper limit, for any decahedral MTPs, the value of K must have a fixed fluctuation range. Predictions of d_A’ and d_B’ for FePd, FeNi, and CoPt intermetallic MTPs were all performed with K=1.012.

References

Chen, W., Yu, R., Li, L., Wang, A., Peng, Q. & Li, Y. (2010). *Angew. Chem. Int. Ed.* **49**, 2917-2921.

Clarke, R. S. & Scott, E. R. (1980). *Am. Mineral.* **65**, 624-630.

Gan, L., Yu, R., Luo, J., Cheng, Z. & Zhu, J. (2012). *J. Phys. Chem. Lett.* **3**, 934-938.

Gautam, A. (2017). Private communication.

Johansson, C. H. & Linde, J. O. (1936). *Ann. Phys.* **417**.

Li, Z.-A., Spasova, M., Ramasse, Q. M., Gruner, M. E., Kisielowski, C. & Farle, M. (2014). *Phys. Rev. B* **89**, 161406.

Momma, K. & Izumi, F. (2011). *J. Appl. Crystallogr.* **44**, 1272-1276.

Ophus, C. (2017). *Adv. Struct. Chem. Imag.* **3**, 13.

Persson, K. (2016).

Warlimont, H. (1959). *Z. Metallkd.* **50**, 708-716.

Woolley, J., Phillips, J. & Clark, J. (1964). *J. Less-Common. Met.* **6**, 461-471.

Yu, Y., Cui, F., Sun, J. & Yang, P. (2016). *Nano Lett.* **16**, 3078-3084.

Yuasa, S., Miyajima, H. & Otani, Y. (1994). *J. Phys. Soc. Jpn.* **63**, 3129-3144.

Material	Type	Single-crystal NPs			Type	MTPs		
		$d_{\boldsymbol{A}}$/nm	$d_{\boldsymbol{B}}$/nm	Angle/°		$d_{\boldsymbol{A}}{}'$/nm	$d_{\boldsymbol{B}}{}'$/nm	Angle/°
Au	Reference	0.2885	0.4080	352.6	Experiment	0.2953	0.4120	356.3
FePt		0.2722	0.3700	363.4		0.2740	0.3770	360.1
AuCu		0.2811	0.3685	373.4		0.2828	0.3884	360.6
CoPt		0.2691	0.3684	361.5	Prediction	0.2707	0.3726	360.0
FeNi		0.2533	0.3582	352.7		0.2618	0.3603	360.0
FePd		0.2716	0.3777	357.2		0.2760	0.3800	360.0

Table 1 Detailed lattice parameters of both original standard single-crystal NPs and MTPs.

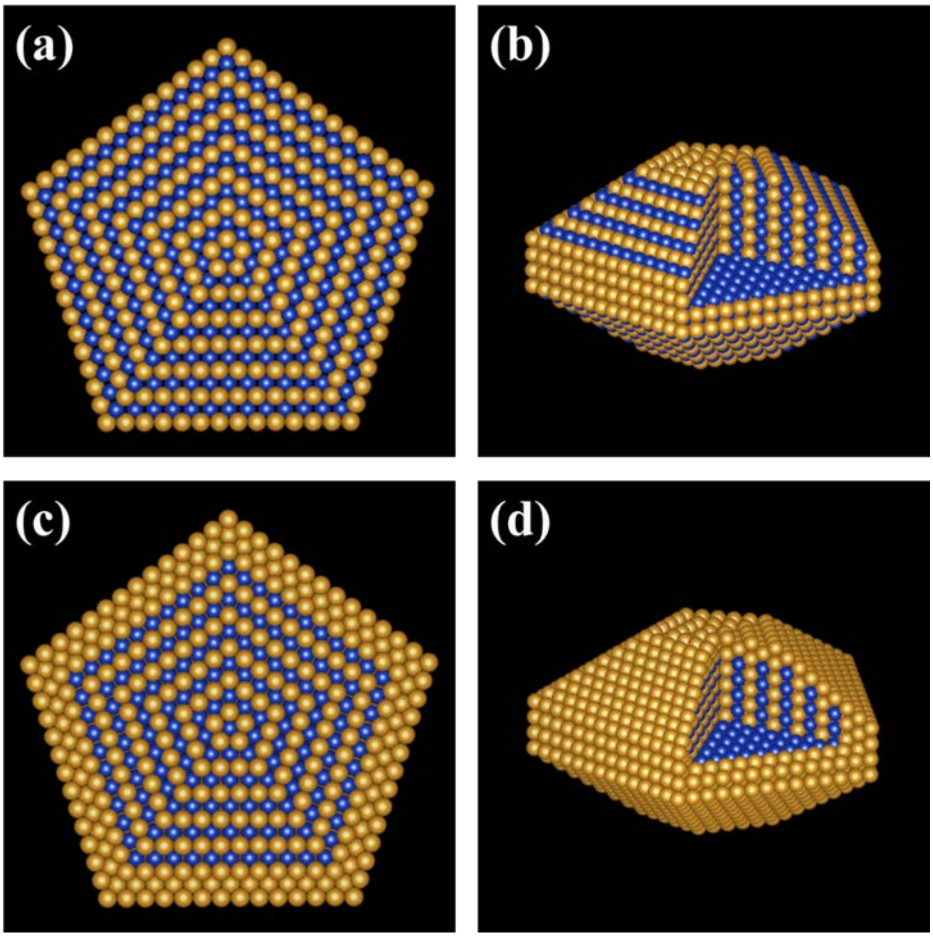

Figure S1 Atomic model of intermetallic AuCu decahedral MTPs. (a) Cross-sectional view and (b) three-dimensional view of the model without gold enriched shell. (c) Cross-sectional view and (d) three-dimensional view of the model with three-layered gold enriched shell.

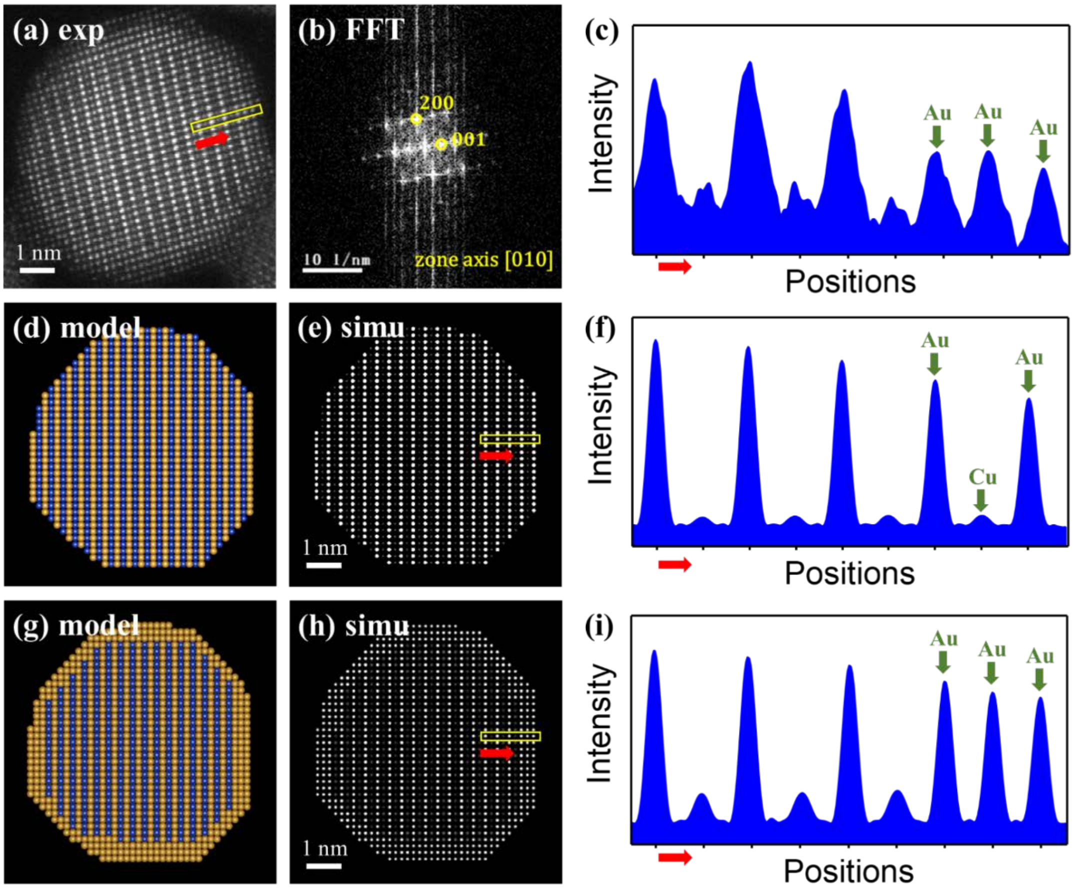

Figure S2 AuCu intermetallic single crystal NPs and surface gold-enriched shell. (a) Experimental atomic resolution HAADF image of an AuCu single crystal NP and its FFT in (b). (c) Intensity profiles across the particle measured along the direction of the red arrow from the yellow box showed in (a). (d) Atomic model of AuCu NP without surface gold-enriched shell, and its simulated HAADF image in (e) and the corresponding intensity profile image in (f). (g) Atomic model of AuCu NP with a surface gold-enriched shell, and its simulated HAADF image in (h) and the corresponding intensity profile image in (i).

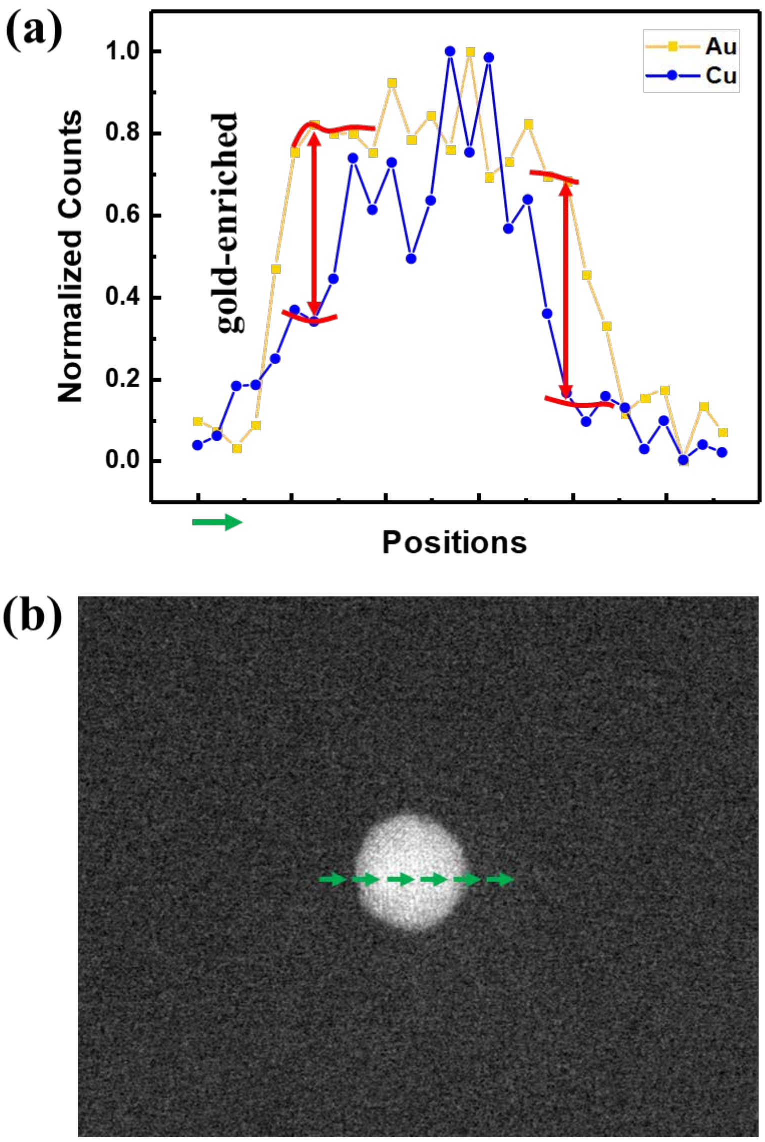

Figure S3 STEM-EELS line profile of an intermetallic AuCu NPs.

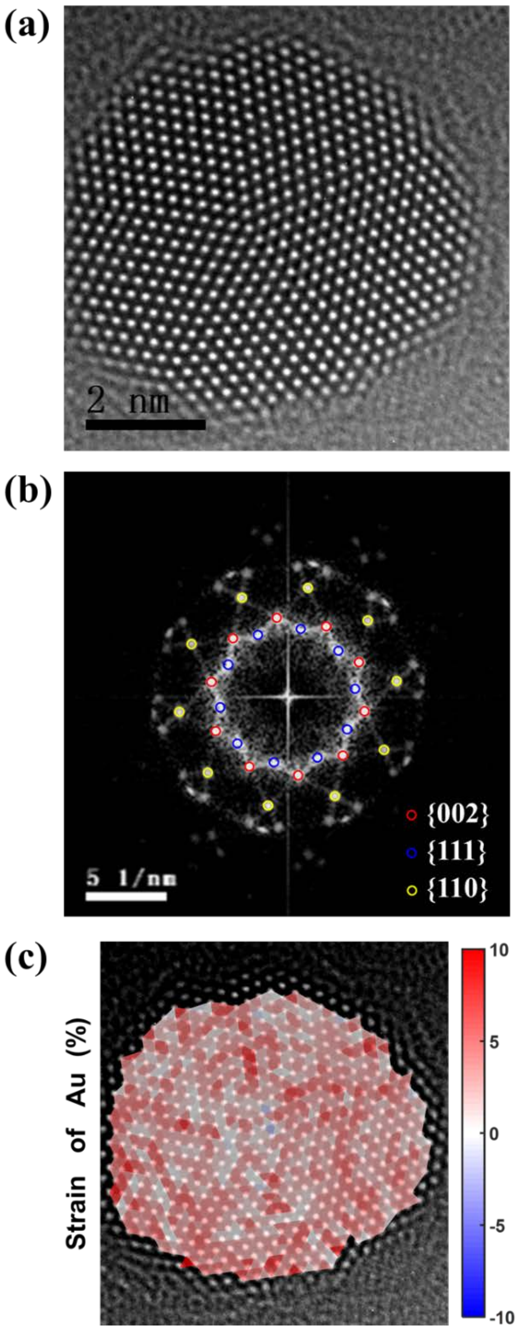

Figure S4 (a) AC-HRTEM image of a gold decahedral MTP along [1$\bar{1}$0] axis. (b) FFT of AC-HRTEM image shown in (a). (c) Two-dimensional lattice strain map of the gold decahedral MTP.

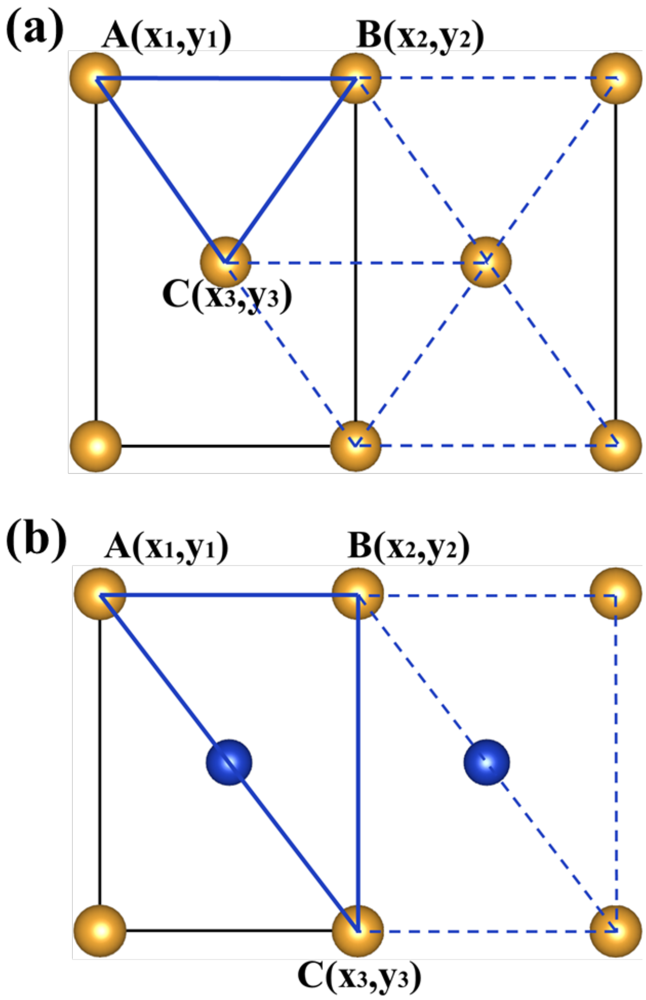

Figure S5 (a) Diagram of the area/strain calculation for Au MTP. (b) Diagram of the area/strain calculation for AuCu MTP.
